# Protocol Paper: Oral Poliovirus Vaccine Transmissibility in Communities After Cessation of Routine Oral Poliovirus Vaccine Immunization

**DOI:** 10.1093/cid/ciy606

**Published:** 2018-10-30

**Authors:** Clea Sarnquist, Marisa Holubar, Lourdes García-García, Leticia Ferreyra-Reyes, Guadalupe Delgado-Sánchez, Luis Pablo Cruz-Hervert, Rogelio Montero-Campos, Jonathan Altamirano, Natasha Purington, Shanda Boyle, John Modlin, Elizabeth Ferreira-Guerrero, Sergio Canizales-Quintero, José Luis Díaz Ortega, Manisha Desai, Yvonne A Maldonado

**Affiliations:** 1 Stanford University School of Medicine, California; 2 Instituto Nacional de Salud Pública, Cuernavaca, Mexico; 3 Bill & Melinda Gates Foundation, Seattle, Washington; 4 Universidad Nacional Autónoma de México (UNAM), Mexico City, Mexico

**Keywords:** poliovirus, oral polio vaccine, inactivated polio vaccine, Mexico, viral transmission

## Abstract

**Background:**

We aimed to elucidate household and community-level shedding and transmission of trivalent oral polio vaccine (tOPV) in communities with inactivated polio vaccine (IPV) routine immunization after tOPV is administered during a national health week (NHW).

**Methods:**

We conducted a 3-arm, randomized trial with data collected at baseline through 10 weeks post-NHW in households with at least 1 child <5 years old in 3 semi-rural communities in Orizaba, Mexico. Selected communities were geographically isolated but socio-demographically similar. Each community was assigned an oral polio vaccine (OPV) immunization rate: 10, 30, or 70% of participating households. From 2653 households in the 3 communities, ~150 households per community were selected, for 466 in total. Households were randomized as vaccinated or unvaccinated, with only 1 child under 5 in the vaccinated household receiving OPV during the February 2015 NHW. No other community members received OPV during this NHW. Stool samples were collected up to 10 weeks post-vaccination for all members of the 466 study households and were analyzed for the presence of OPV serotypes using a multiplex polymerase chain reaction assay.

**Results:**

We will report on the factors associated with, and incidence and duration of, household and community shedding and transmission of OPV. The secondary outcomes will characterize temporal and geospatial OPV serotype shedding patterns.

**Conclusions:**

The current global polio eradication plan relies on transitioning away from OPV to IPV. This study contributes to understanding patterns of OPV shedding and transmission dynamics in communities with primary IPV immunity, in order to optimize the reduction of OPV transmission.

The World Health Assembly declared in 2012 that polio eradication constitutes “a programmatic emergency for global public health,” and released The Polio Eradication and Endgame Strategic Plan 2013–2018, which aims to both eradicate wild poliovirus and eliminate circulating vaccine-derived poliovirus (cVDPV) [1]. The oral Sabin-type polio vaccine (OPV) has been the primary vaccine used for prevention in developing settings, but it acquires point mutations that can cause vaccine-associated paralytic poliomyelitis (VAPP). VAPP occurs in 1 in 2.7 million first OPV doses and 1 in 51.6 million subsequent doses in the Latin American population [2]. In addition, OPV may continue to be transmitted, primarily in under-immunized communities, and over time can mutate into cVDPVs, which have reacquired neurovirulence. Thus, the elimination of both VAPP and cVDPVs are essential for polio disease eradication. The World Health Organization’s Strategic Plan has established that successfully eliminating cVDPVs depends on the final withdrawal of all OPV vaccines by 2020. Withdrawal began in 2016 with removal of the serotype 2 component (OPV-2) of trivalent OPV (tOPV), due to the declaration of the elimination of serotype 2 wild-type poliovirus in 2015. OPV-2 withdrawal involves strengthening of countrywide immunization systems, as it requires that at least 1 dose of inactivated poliovirus vaccine (IPV) be incorporated into routine vaccination programs, as well as requires the removal and replacement of tOPV with bivalent OPV (bOPV) in all countries that utilize OPVs.

In order to guide decision-making around the timing of OPV use and cessation, several important questions remain to be answered. Limited data are available on household and community transmission of OPV in settings where both OPV and IPV are in use concurrently. Understanding how immunization with both OPV and IPV—as well immunities elicited individually by OPV and IPV—might affect OPV transmission and circulation patterns is vital for the eventual eradication of OPV, VAPP, and cVDPV. This knowledge will help inform OPV cessation strategies, as well as ongoing vaccination and surveillance needs. Our research group has previously shown that OPV may circulate for up to 3, 4, or 5 months after each episode of OPV vaccination in communities receiving both IPV and OPV, allowing the possibility of persistent OPV transmission and disease [3]. Further elucidation of these complicated relationships is essential to make optimized decisions about when and how to decrease, and eventually cease, OPV use in favor of IPV. Additionally, policy regarding how to conduct human and environmental surveillance for OPV and cVDPV would also be informed by understanding the natural history of OPV shedding and transmission in populations with varying levels of immunization.

The present study aims to understand household and community OPV transmission in Mexico, especially as a function of variable OPV vaccination rates. Mexico provides a unique natural environment for such a study because, as of early 2007, the Mexican national vaccination program was among the few country programs providing routine IPV, with OPV administered only during twice annual national health weeks (NHWs).

## METHODS/DESIGN

**Figure 1.**
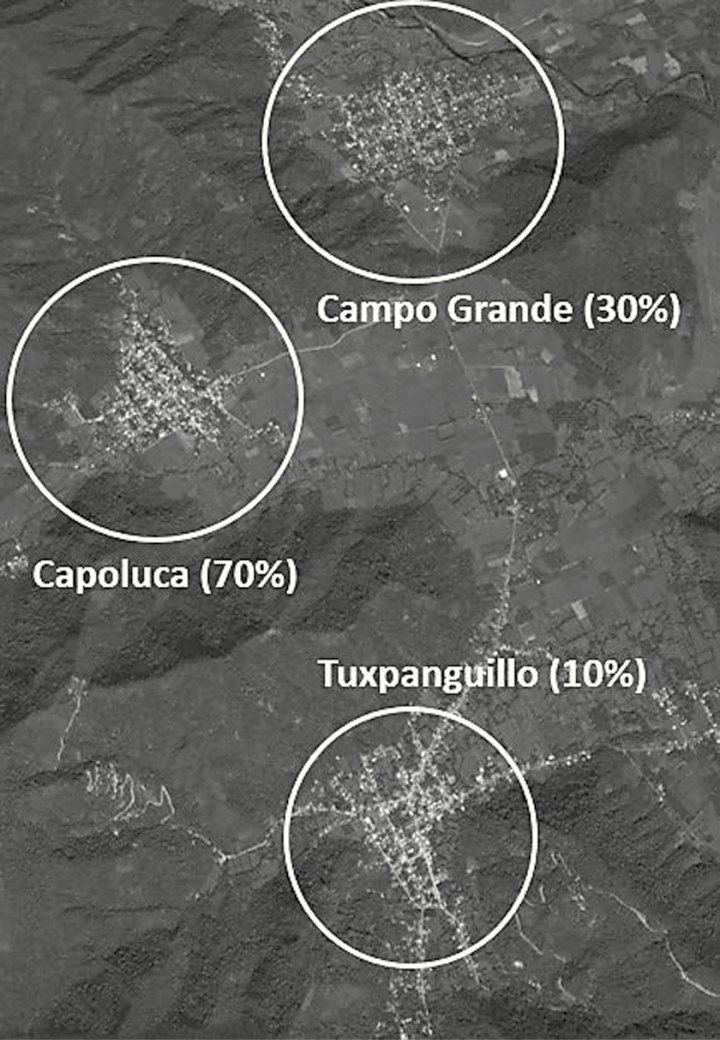
Map of study communities and vaccination rate.

We conducted a 3-arm community study in ~450 households in 3 communities of the semi-rural Ixtaczoquitlán municipality in Orizaba, Veracruz, Mexico. The selected communities were Tuxpanguillo, Campo Grande, and Capoluca (Figure 1). Each community was assigned an OPV vaccination rate of either 10%, 30%, or 70%.

Sampled households had at least 1 child under 5 and the families were randomized into either an OPV-vaccinated or unvaccinated household during the February 2015 NHW. These vaccination rates were chosen to proxy various possible community vaccination rates, with the 10% and 30% communities representing low to moderate levels of vaccination coverage, whereas the 70% community might be considered a moderately high vaccination coverage rate. Households were eligible to participate in the study if they resided in one of the 3 communities and had at least 1 healthy child under 5 in the household who was up to date with her/his complete polio virus vaccination schedule as follows: (1) 8 months to 16 months and 29 days of age and had received 3 doses of documented IPV, or (2) 20 months to 4 years, 11 months, and 29 days with 4 doses of documented IPV. Children who were ineligible due to poor health included those presenting with acute respiratory infection, loose bowel movements, primary immunodeficiencies, or AIDS symptomatology; with hemato-oncologic diseases or other neoplasias; receiving treatment with corticosteroids and other immunosuppressants or cytotoxic medicines; having presented a severe adverse reaction to previous OPV doses; or with a history of having received gammaglobulin or a blood transfusion in the 3 months prior to the date of vaccination. The 3 localities were selected for their similarities in sociodemographic, epidemiological, health, cultural, and health access characteristics. The communities are all located in a relatively mountainous area and also physically separated from each other by valleys, helping assure minimal OPV transmission from 1 community to the next and allowing us to largely isolate transmission within each community.

### Census and Randomization

**Figure 2.**
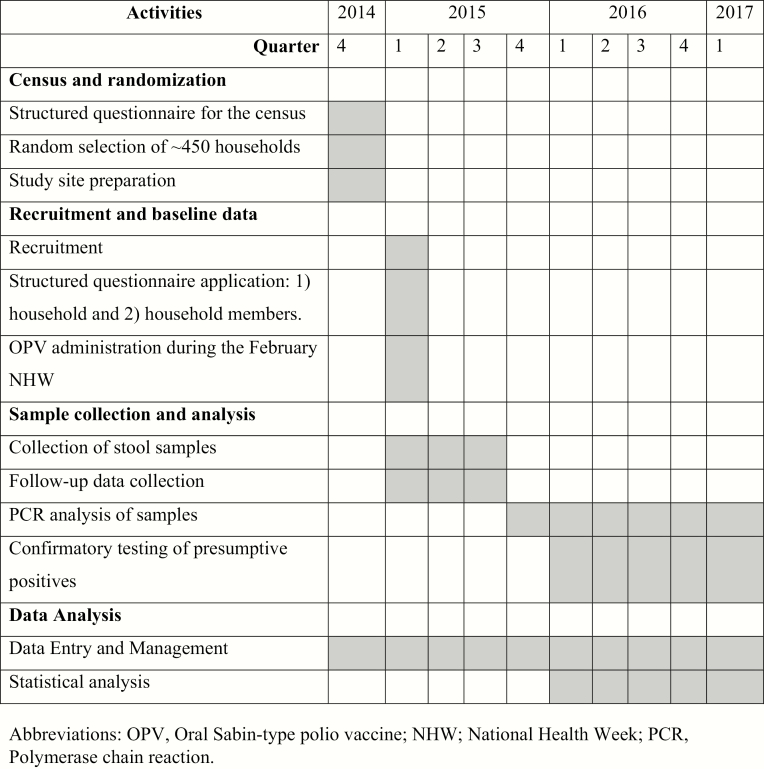
Study timeline.

The study began with a census of every household in each community; a structured questionnaire was administered to all household members in the 3 communities (see Figure 2 for entire timeline). This questionnaire ascertained household characteristics, economic characteristics, household density, number of children younger than 5 years of age, and child vaccination schedules, which were verified, when possible, by vaccination cards (Table 1). In conjunction with the census survey, study team nurses also conducted vaccine promotion activities in all households, in an effort to encourage household parents or guardians to bring their under-5 children to each community’s health center and ensure each child’s vaccination schedules were up to date. When required, transportation to and from the health centers was also provided to families. As part of the census, all residences where a child younger than 5 years of age had been surveyed were geo-referenced using handheld geospatial positioning devices.

**Table 1.** Key Census Variables, by Community

	Capoluca	Campo Grande	Tuxpanguillo	Total
Number of families	740	834	1079	2653
Total population	2904	3299	4287	10490
Number of men (%)	1448 (49.9%)	1591 (48.2%)	2098 (48.9%)	5137 (49%)
Number of women (%)	1456 (50.1%)	1708 (51.8%)	2189 (51.1%)	5353 (51%)
Number of children under 5 years (%)	301 (10.4%)	310 (9.4%)	415 (9.7%)	1026 (9.8%)
Mean age, in years	26.8	28.3	28.5	28
Mean family size	3.9	4.1	4	3.96
Number of families with father’s religion as Catholic (%)	696 (94.1%)	791 (94.8%)	956 (88.6%)	2443 (92.1%)
Number of families with mother’s religion as Catholic (%)	697 (94.1%)	795 (95.3%)	960 (89.0%)	2452 (92.4%)
Number of children under 5 on schedule for inactivated polio vaccine (%)	273 (90.7%)	239 (77.1%)	384 (92.5%)	896 (87.3%)
Number of houses with running water (%)	601 (81.2%)	668 (80.1%)	972 (90.1%)	2241 (84.5%)
Number of houses with toilets (%)	520 (70.3%)	652 (78.2%)	971 (90.0%)	2143 (80.8%)
Number of houses with electricity (%)	709 (95.8%)	817 (98.0%)	1062 (98.4%)	2588 (97.5%)

**Figure 3.**
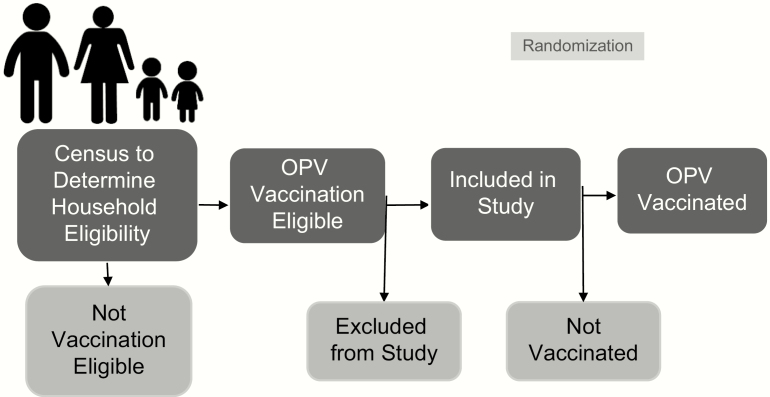
Sampling and vaccination scheme. Abbreviation: OPV, oral polio vaccine.

Communities were assigned randomly to a percentage of study participants (children under 5 in eligible households that were selected for study participation) that would receive the OPV vaccination, with 10% in Tuxpanguillo, 30% in Campo Grande, and 70% in Capoluca receiving the vaccine (Figure 3). Once ~150 households eligible for the study were identified, those households in which 1 child under 5 years of age was vaccinated with OPV were randomly selected using a random number generator.

### Recruitment

Study nurses visited all of the households in the 3 communities that had a child under 5 but were not sampled to participate in the study. In those households, study nurses requested that a parent or legal guardian sign an Informed Consent Letter to allow their children to not receive OPV during the February NHW. Thus, during the 2015 NHW week, the only children in each of the municipalities to receive OPV were those randomly sampled to participate in the vaccination. However, other normal NHW activities, such as Vitamin A supplementation and nutrition education, took place in all households within the communities. Furthermore, all children who were randomized to not receive OPV during the NHW were up-to-date on their IPV vaccination schedules and were encouraged to receive an OPV at the May 2015 NHW.

### Stool Samples and Follow-up

**Figure 4.**
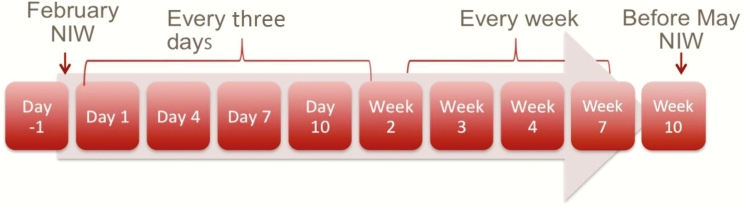
Stool sample collection timeline. Abbreviation: NIW, National Immunization/Health Week.

Beginning 1 day before the first NHW started, and for 71 days after (see Figure 4), each household member for all of the ~150 sampled households in each community (vaccinated and un-vaccinated) provided stool samples to be tested for OPV. Stool samples collection was planned from all members of the sampled households on days -1, 1, 4, 7, 10, 14, 21, 28, 60, and 90, for a total of 10 samples per person. However, since samples could not be collected after the May NHW began, and that NHW ended up being planned for earlier than originally thought, the last 2 collection points were changed to occur at 51 and 71 days, instead of at 60 and 90 days. When sample collection was scheduled for every third day (samples 2 to 6), there was a margin of 1 day prior or subsequent to the date to collect the sample. When sample collection was scheduled to be taken weekly (samples 7 to 9), there was a margin of 3 days after the date to collect the sample. For the collection of the last planned sample (sample 10), there was a margin of 7 days after the date to collect the sample. Families that moved out of the geographic study region during the follow-up period were excluded from follow-up.

### Stool Sample Analysis

The poliovirus detection assay was developed specifically for this study and was a multiplex 2-step reverse transcription, quantitative polymerase chain reaction assay (RT-qPCR). Ribonucleic acid was extracted from frozen stool samples using the MagMAX Viral RNA Isolation Kit on the Kingfisher Duo Prime instrument (both from Thermo Scientific Inc.), and reverse transcription utilized the SuperScript III Reverse Transcriptase (Thermo Fisher Scientific Inc.). Real-time polymerase chain reaction was performed in a total volume of 20 microliters, consisting of 5 microliters of complementary deoxyribonucleic acid (cDNA) and 15uL of iQ Multiplex Powermix with Sabin 1, 2, and 3 specific primers and probes.

Amplification and detection was performed on the CFX384 Real-Time System (BioRad). Samples were run in triplicate, and a sample was considered positive if 2 out of 3 reactions crossed the cycle threshold (C_T_) in less than 37 cycles. To account from sample handling variability during real-time polymerase chain reaction testing, Sabin 1, 2 and 3 positive controls, a negative control, and No Template Control (NTC) were then run together on 384 well plates with the unknown samples above.

Finally, genetic sequencing using the Sanger technique was performed on these OPV-positive samples to rule out the possibility of false positives due to other enteroviruses [4].

### Data Management and Analysis Plan

Data were double-entered into a SQL database and underwent primary cleaning on site in Mexico. After primary cleaning, the data were exported into a CSV file and sent to Stanford. This CSV file was then uploaded to a FileMaker Pro database, where data cleaning was finalized and data were linked together via family and subject identification numbers, as well as linked with laboratory results.

We conducted 3 specific analyses in order to address the primary objectives of incidence and duration of household and community shedding and transmission of OPV. First, to examine overall shedding, logistic regression models were fit to shedding at any point in the study as a function of locality, household vaccination status, and their interaction. The significance of the interaction term determined whether transmission differed in each locality by household vaccination status. Second, to assess these same associations over time, longitudinal logistic models were fit to shedding/transmission at a certain time as a function of locality, household vaccination status, time in days, quadratic time (only for the “within vaccinated household” models), and the interaction between locality, household vaccination status, and each time variable. The significance of the joint test of both interaction terms determined whether the trajectory of shedding was different in each locality by household vaccination status. Finally, to determine whether time to first shedding differed across locality and vaccination status, Cox proportional hazards models were fit to time to first shedding as a function of locality, household vaccination status, and their interaction.

Due to subject clustering within families and repeated measures on subjects over time, the marginal model approach using an exchangeable correlation structure was utilized to address our research questions. This approach is generally used in the analysis of correlated data, adjusting for the correlation by robust sandwich estimates [5]. Failing to account for possible correlations can lead to underestimation of the variance, which can result in artificially low *P* values. Therefore, to account for household clusters, a household cluster effect was included in the overall shedding models, as well as the Cox models. To account for household clusters and repeated measures on subjects over time, a subject-nested-in-household cluster effect was included in the longitudinal logistic models. All analyses were conducted for overall OPVs, including by serotype. The analyses were further adjusted for continuous age, whether or not the house had a running toilet, and household density (number of people living in the house). For the within-household analyses, odds/hazard ratios and 95% confidence intervals were reported for household contacts versus vaccinated children and for each pairwise comparison within household contacts. For the community analyses, odds/hazard ratios and 95% confidence intervals were reported for household contacts versus unvaccinated household participants and for each pairwise comparison within unvaccinated household participants. A *P* value of <.05 was considered statistically significant. All analyses were conducted using SAS statistical software, version 9.4 (SAS Institute, Inc., Cary, NC).

## ETHICS

This study was reviewed and approved by the Stanford University Institutional Review Board (Protocol #31546); the Comité de Etica, Bioseguridad e Investigación of the Instituto Nacional de Salud Pública (CI: 1260, No. 1581); and the Instituto Veracruzano para la Formación e Investigación en Salud (SESVER/IVEFIS//SIS/DIB/0109/02014, classification 15S). The study is registered with ClinicalTrials.gov (#34706).

## DISCUSSION

This study aimed to fill gaps in the current knowledge about poliovirus circulation in order to better understand the impact of persistent shedding and transmission of OPVs on global polio disease eradication. Findings from this study will help elucidate the OPV circulation patterns when live poliovirus is re-introduced in an area with primary, IPV-induced immunity. The study should also provide an understanding of factors which could impact household and community shedding and transmission of OPVs, such as previous exposure to IPV, number of previous IPV doses, age, distance from vaccinated household, and others. Additional information regarding geospatial patterns of OPV shedding and transmission were also analyzed. These communities in periurban Mexico provided a unique opportunity to examine the interaction between IPV immunity and OPV transmission as, at the time of inception of the study, Mexico was among the few countries with both routine IPV immunization as well as twice-annual OPV immunization. These data will help inform the safe global use of OPVs as IPV becomes the primary immunization approach.

A key area of new information this study provides is in the role of combined IPV and tOPV immunization on viral shedding, and therefore mucosal immunity, in communities. Because IPV protects against paralytic poliomyelitis, but only offers limited mucosal protection against wild-type and vaccine-derived polio virus infections, poliovirus can continue circulating even within a community with high IPV coverage rates, as well as be transmitted to communities that have low vaccination rates due to religious preferences or political realities [6, 7]. Recent evidence suggests that while IPV vaccination alone does not provide mucosal immunity, IPV vaccination after tOPV vaccination may significantly boost mucosal immunity, especially if 2 doses of IPV are given [8, 9]. The finding that IPV may strengthen the protection provided by OPV has been replicated in a large, randomized, controlled trial with bOPV; specifically, infants receiving bOPV as well as 1 or 2 doses of IPV seroconverted 80 and 100% of the time, respectively [10]. Intestinal immunity was also demonstrated in many of these infants. It has further been shown that the amount of virus shed is influenced by mucosal immunity [11]. In addition, it has been shown that IPV doses boost immunity in children with malnutrition, a population of particular importance since the countries with remaining polio, or recent clinical outbreaks, all have areas where malnutrition is a significant problem [12]. Thus, the information the current study provides on viral circulation patterns in communities where both IPV and OPV are in use may be especially relevant for eradication efforts.

Another area where this study fills data gaps is in understanding the community and geospatial spread of polio. Data on community spread of vaccine-derived poliovirus suggest about a 20% community transmission rate, but are mostly from the United States and from the 1950’s and 1960’s; thus, there is a gap in the current understanding [13, 14]. Geographic mapping of transmission is more recent, but has generally happened over larger areas rather than within defined communities, and often has been predictive for the purpose of outbreak control, such as for the recent outbreak in Nigeria [15]. The data from this study provides a unique look at how real (non-theoretical) poliovirus shedding moves within communities over time.

Finally, this data provides an opportunity to analyze viral loads in comparison to shedding over time, by serotype, and across the different communities. Although a few other studies have measured viral loads, this measurement has previously been incorporated into larger viral indices rather than being considered as a separate variable that is likely linked to transmission [10, 16].

### Limitations

There are 4 major limitations of this study. First, no information was collected on either baseline or post-vaccination serum poliovirus antibodies in the study population. However, vaccination histories obtained from National Vaccination cards were utilized in the cases of minors less than 5 years of age or from self-reports in those over 5 years of age as an approximation of the prior immune status of the study participants. Second, oropharyngeal shedding of OPV was not measured. However, it has been documented that, in medium- and low-resource regions, such as with this study’s populations, fecal shedding is more prominent than oropharyngeal shedding. Third, the vaccine virus may have been introduced from visits with vaccinated children from other regions. Fourth, individuals who resided in the households that did participate in OPV vaccinations during the NHW may have decided to be vaccinated elsewhere. In order to control for this possibility in the analysis, each follow-up visit included questions about changes in vaccination status in order to capture the frequency of this decision.

## CONCLUSION

This study is among only a handful, many of which were performed decades ago, to study OPV transmission at the community level, as well as being among the very few in environments that mimic those required by the Polio Eradication and Endgame Strategy, where OPV and IPV are used concurrently during a controlled switchover. Thus, it provides essential data to inform global polio eradication efforts.

## Notes

***Acknowledgments.*** The authors thank the families who participated in this study, the large field teams in Mexico that performed data and sample collection, and the broader team at Stanford University, including Christopher Anthony van Hoorebeke, Sean Leary, Aisha Talib, Jennifer Lee Kang, Rasika Behl, Meira Falkovitz-Halpern, ChunHong (Stacy) Huang, and Marvin Sommer.

***Disclaimer.*** The sponsor was an active partner in the study design but was not involved in the data collection, analysis, and interpretation, nor in the writing of the report or the decision to submit this article for publication.

***Financial support.*** This work was supported by the Bill & Melinda Gates Foundation (grant number OPP1116910).

***Supplement sponsorship.*** This article appears as part of the supplement “Polio endgame and beyond: Vaccine choices, transmission dynamics, and surveillance implications,” sponsored by The Bill & Melinda Gates Foundation.

***Potential conflicts of interest.*** S. C.-Q., E. F.-G., L. P. C.-H., G. D.-S., L. F.-R., L. G.-G., and R. M.-C. received grants and personal fees from the BMGF and Stanford University during the conduct of the study and grants and non-financial support from GlaxoSmithKline Biologicals outside the submitted work. J. L. D.-O. received grants from the BMGF and Stanford University during the conduct of the study. Y. A. M. received grants from the BMGF during the conduct of the study and personal fees and other from Pfizer outside the submitted work. N. P., C. S., J. A., and M. H. received grants from the BMGF during the conduct of the study. All other authors report no potential conflicts of interest. All authors have submitted the ICMJE Form for Disclosure of Potential Conflicts of Interest. Conflicts that the editors consider relevant to the content of the manuscript have been disclosed.
